# Regulation of aromatase expression in the anterior amygdala of the developing mouse brain depends on ERβ and sex chromosome complement

**DOI:** 10.1038/s41598-017-05658-6

**Published:** 2017-07-13

**Authors:** Carla Daniela Cisternas, Lucas Ezequiel Cabrera Zapata, María Angeles Arevalo, Luis Miguel Garcia-Segura, María Julia Cambiasso

**Affiliations:** 1 Instituto de Investigación Médica Mercedes y Martín Ferreyra, INIMEC-CONICET-Universidad Nacional de Córdoba, Córdoba, Argentina; 2 Departamento de Biología Bucal, Facultad de Odontología -Universidad Nacional de Córdoba, Córdoba, Argentina; 3 Instituto Cajal, CSIC, Madrid, Spain; 4 Ciber de Investigación Biomédica en Red de Fragilidad y Envejecimiento Saludable (CIBERFES), Instituto de Salud Carlos III, Madrid, Spain

## Abstract

During development sex differences in aromatase expression in limbic regions of mouse brain depend on sex chromosome factors. Genes on the sex chromosomes may affect the hormonal regulation of aromatase expression and this study was undertaken to explore that possibility. Male E15 anterior amygdala neuronal cultures expressed higher levels of aromatase (mRNA and protein) than female cultures. Furthermore, treatment with oestradiol (E2) or dihydrotestosterone (DHT) increased *Cyp19a1* expression and aromatase protein levels only in female neuronal cultures. The effect of E2 on aromatase expression was not imitated by oestrogen receptor (ER) α agonist PPT or the GPER agonist G1, but it was fully reproduced by DPN, a specific ligand of ERβ. By contrast, the effect of DHT on aromatase expression was not blocked by the anti-androgen flutamide, but completely abrogated by the ERβ antagonist PHTPP. Experiments using the four core genotype model showed a sex chromosome effect in ERβ expression (XY > XX) and regulation by E2 or DHT (only XX respond) in amygdala neurons. In conclusion, sex chromosome complement governs the hormonal regulation of aromatase expression through activation of ERβ in developing mouse brain.

## Introduction

The field of sexual differentiation has been receiving increasing contributions since the pioneering work of Phoenix *et al*.^1^. The noteworthy evidence that testosterone (T) influences sex-specific behaviours through organisation of the developing brain during a critical period early in development^1^, complemented with later evidence that oestradiol (E2) is an important metabolite in the brain^2^ has established the basis for the latter studies. More recently sex chromosome genes have been recognized as the primary factors causing sexual differentiation (reviewed in Arnold, 2017^3^) not only as direct effectors of some sex specific traits but also acting in parallel with sex steroids. Aromatase, one of the last enzymes of neurosteroidogenesis, is responsible for the conversion of T into E2. The expression of aromatase is limited to sexually dimorphic regions of the brain^4–6^ and presents sex differences during the sensitive period of sexual differentiation^4^.

In concordance with previous results we recently demonstrated a sex chromosome-induced difference in aromatase expression in the anterior amygdaloid area and the stria terminalis of the mouse brain as early as embryonic day 16^7^ (E16). These and previous findings of higher activity and expression of aromatase in hypothalamic regions of male rat and mouse brain at E16^8–11^ are not determined by the prenatal increase in T production by the foetal testis, which in rats occurs at E18.5–19.5^12, 13^ and in mice at E17–18^14, 15^. Importantly, the use of the “Four Core Genotypes” (FCG) mouse model in our previous study^7^ allowed us to establish that sex chromosomes influence aromatase independently of gonadal status. This mouse model combines a spontaneous deletion of the sex determining region Y (*Sry* gene) from the Y chromosome (Y^−^) with the re-insertion of a functional *Sry* transgene onto an autosome^16, 17^. The XY^−^ *Sry* mice have testes and are fully fertile. The *Sry* transgene and the Y^−^ chromosome separate independently during meiosis, thus, four genotypes are present in the offspring: XX females, XY^−^ females, XX*Sry* and XY^−^ *Sry* male mice.

The regulation of aromatase expression by gonadal steroids has been well documented^18–22^. While some evidence indicates that androgens and oestrogens synergize to induce aromatase mRNA in the quail brain^23^, other evidence indicates that dihydrotestosterone (DHT) seems to be more effective than T in the induction of aromatase^21^. Importantly, in well-known sexually dimorphic brain regions such as the preoptic area and hypothalamus, testosterone and DHT induce aromatase expression^24^. Our previous study using amygdala neuronal cultures indicates that E2 and DHT regulate aromatase expression through a mechanism controlled by sex chromosome complement. E2 and DHT increase aromatase mRNA levels in cultures of anterior amygdala neurons coming from XX embryos^7^. Since oestrogen (ER) and androgen (AR) receptors are critical for steroid hormone effects during brain development and their expression mostly corresponds to brain regions which express aromatase^25^, brain masculinization requires ERs in some brain regions and AR function in others. In addition, although ERα has been implicated in the masculinizing actions of oestradiol in hypothalamic and preoptic areas^26^ less is known about ERβ involvement during development.

In the present study we evaluated whether sex chromosome factors determine the mechanism by which sex hormones regulate aromatase expression in the amygdala. Our findings indicate that differential expression of ERβ is regulated by sex chromosomes, and underlies the hormonal regulation of neuronal aromatase expression in the amygdala. Given that this study was carried out before the critical period of increased gonadal secretion, these findings imply that genetically controlled mechanisms precede gonadal influences during the genesis of differences between the sexes in brain structure.

## Results

### Male neuronal cultures express higher levels of aromatase (mRNA and protein) than female cultures

**Figure 1:**
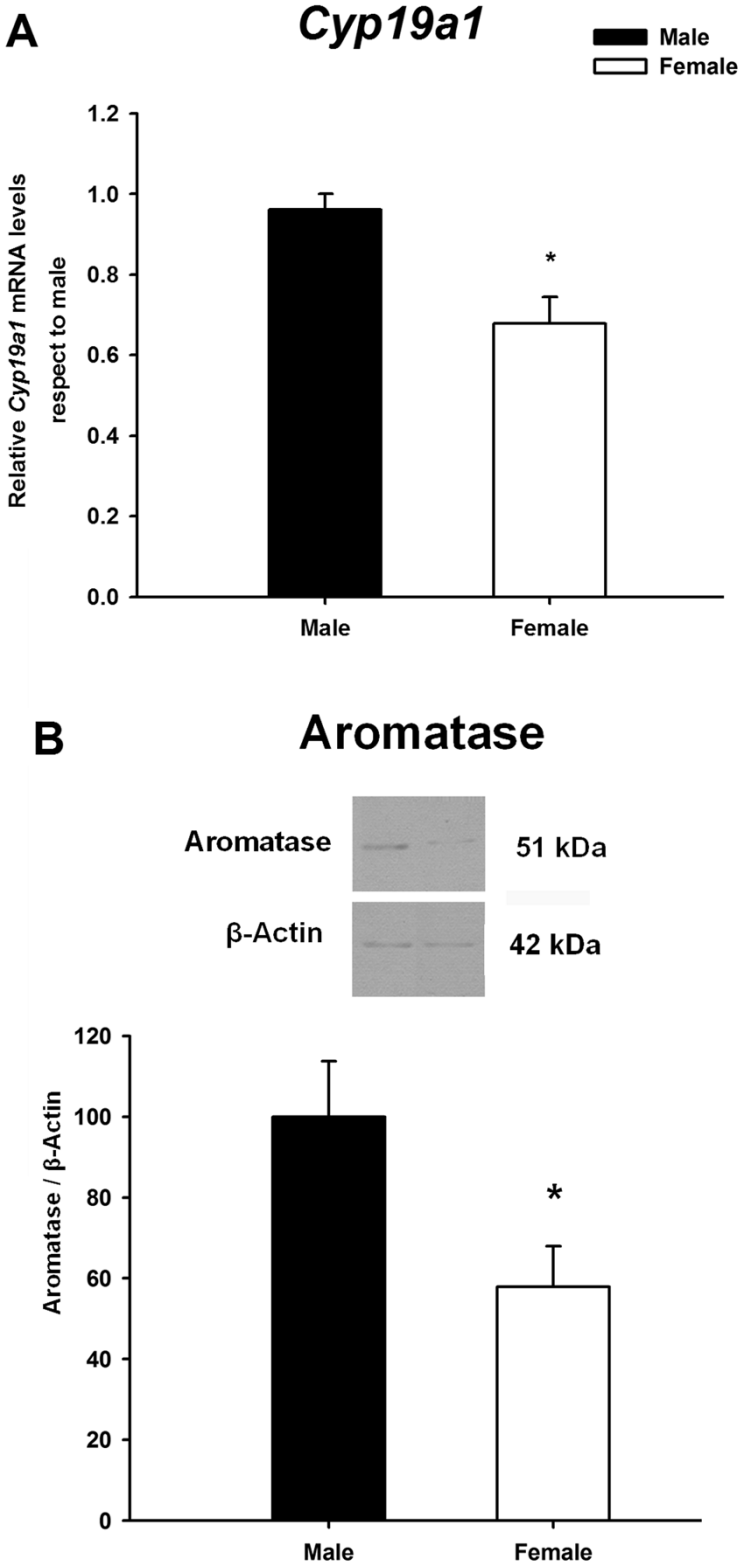
Expression of aromatase mRNA (*Cyp19a1*, **A**) and protein (**B**) in male and female primary amygdala neuronal cultures from CD1 mouse embryos. Male cultures had higher expression of aromatase mRNA and protein levels than females. *p ≤ 0.05. Bars indicate mean + SEM. *n* = 4–6 independent cultures.

In agreement with previous studies in the anterior amygdala *in vivo* ^7^, male neuronal cultures of E15 CD1 embryos expressed higher levels of *Cyp19a1* mRNA (Student’s t-test: p = 0.05; Fig. 1A) and this was also the case for protein expression (Student’s t-test: p = 0.04; Fig. 1B).

### Oestradiol increases *Cyp19a1* expression and aromatase protein levels only in female neuronal cultures by a mechanism involving ERβ

**Figure 2:**
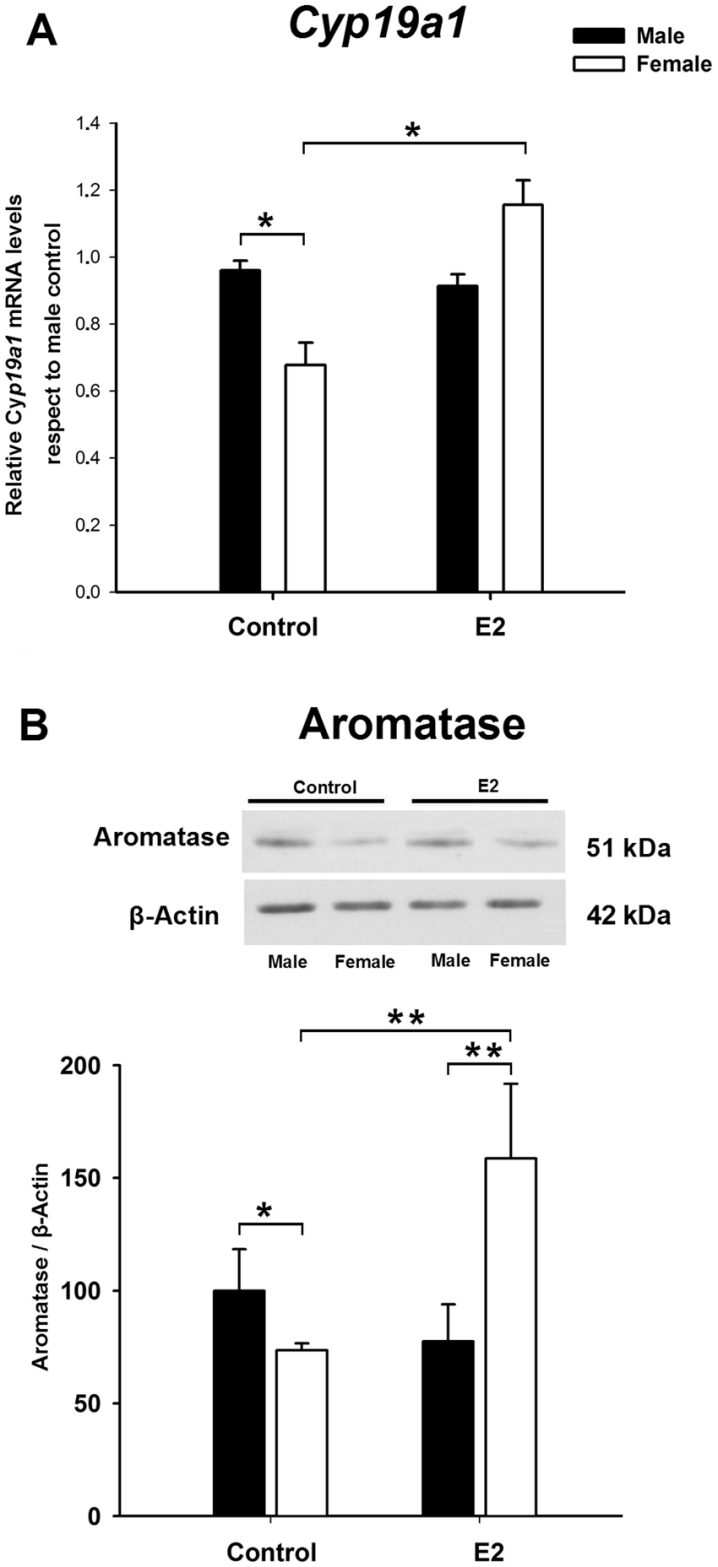
Effect of 17β-oestradiol (E2) on aromatase mRNA (*Cyp19a1*, A) and protein (B) in male and female primary amygdala neuronal cultures from CD1 mouse embryos. E2 stimulation increased aromatase mRNA levels in female but not in male neuronal cultures. *p ≤ 0.05 vs. male and female + E2. Bars indicate mean + SEM. *n* = 4–6 independent cultures.

To determine whether E2 affects aromatase expression in a sex-specific manner, male and female amygdala cultures were treated with the hormone or vehicle. E2 treatment resulted in a significant increase in *Cyp19a1* expression only in female cultures (interaction of sex by treatment for *Cyp19a1* expression: F_1,23_ = 4.94; p = 0.036; Fig. 2A). *Cyp19a1* expression levels in E2-treated female cultures were comparable to the levels in male control cultures (LSD test: p = 0.60). Similar results were obtained for aromatase protein levels. Two-way ANOVA revealed a significant sex-by-treatment interaction (F_1,17_ = 11.97; p = 0.002; Fig. 2B). In neuronal cultures originating from female embryos E2 treatment resulted in an increase of aromatase protein levels (LSD test: p = 0.005) and in the abolishment of sex differences between E2-treated female cultures and control male cultures (LSD test: p = 0.26). On the other hand, E2 exposure did not modify aromatase protein levels in males (p = 0.12).

**Figure 3:**
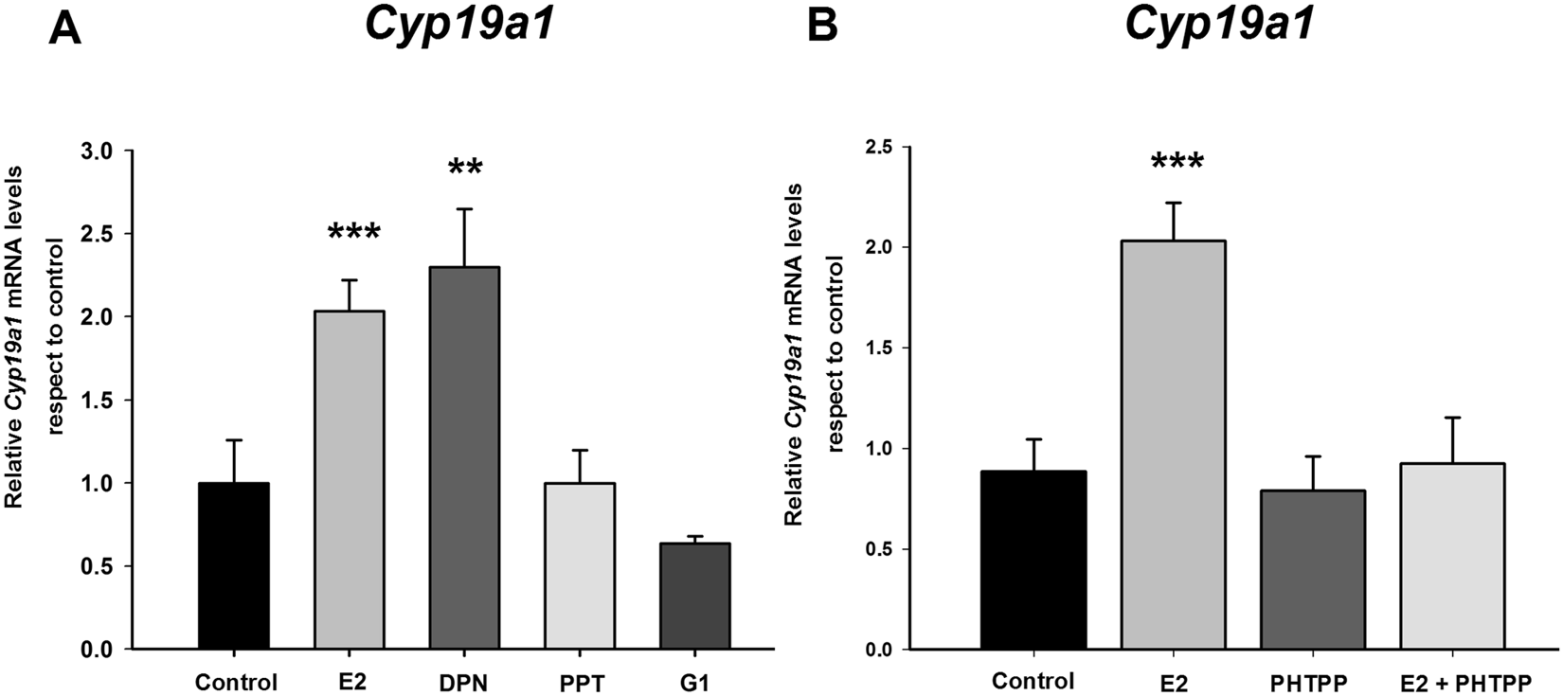
Effect of selective agonists of oestrogen receptors (**A**) and selective ERβ antagonist (**B**) on E2-induced aromatase mRNA (*Cyp19a1*) expression in amygdala neuronal cultures from female CD1 mouse embryos. ERβ agonist DPN imitated the effect of E2 on *Cyp19a1* and PHTPP (ERβ antagonist) prevented this effect. **p < 0.01, ***p < 0.001. Bars indicate mean + SEM. n = 4–6 independent cultures. PPT: selective agonist of ERα, G1: selective agonist of GPER.

In order to identify the ER subtype involved in the regulation of *Cyp19a1* by E2, female neuronal cultures were treated with agonists for ERα, ERβ or G protein-coupled oestrogen receptor 1 (GPER). One-way ANOVA showed a significant main effect of treatment (F_4,21_ = 14.10; p < 0.0001; Fig. 3A). LSD test demonstrated that only the selective ERβ agonist DPN was able to imitate the effect of E2, causing a significant increase in *Cyp19a1* levels (LSD test: p < 0.0001). The treatment of female cultures with the ERα agonist PPT or the GPER agonist G1 did not significantly affect *Cyp19a1* levels (p = 0.77 and p = 0.20 respectively). We further examined the effect of E2 in combination with the selective ERβ antagonist PHTPP. One-way ANOVA demonstrated a main effect of treatment (F_3,14_ = 11.58; p < 0.001), and PHTPP blocked the effect of E2 on *Cyp19a1* levels (LSD test: p = 0.00059; Fig. 3B). Furthermore, PHTPP alone did not affect *Cyp19a1* expression (LSD test: p = 0.71).

### 3β-diol mediates the effect of DHT on aromatase expression in female neuronal cultures by a mechanism involving ERβ

Our previous results showed the expression of 5α-reductase enzymes I and II in punches of the amygdala from E16 male and female mouse embryos. Therefore, the amygdala has the enzymatic machinery to synthetize DHT from T. In addition, we have shown that the treatment with DHT resulted in an increase in *Cyp19a1* mRNA levels in amygdala neuronal cultures originating from E15 female mice^7^. In view of this finding, the same experimental procedure was applied to amygdala neuronal cultures in order to determine the mechanism involved in this regulation.

**Figure 4:**
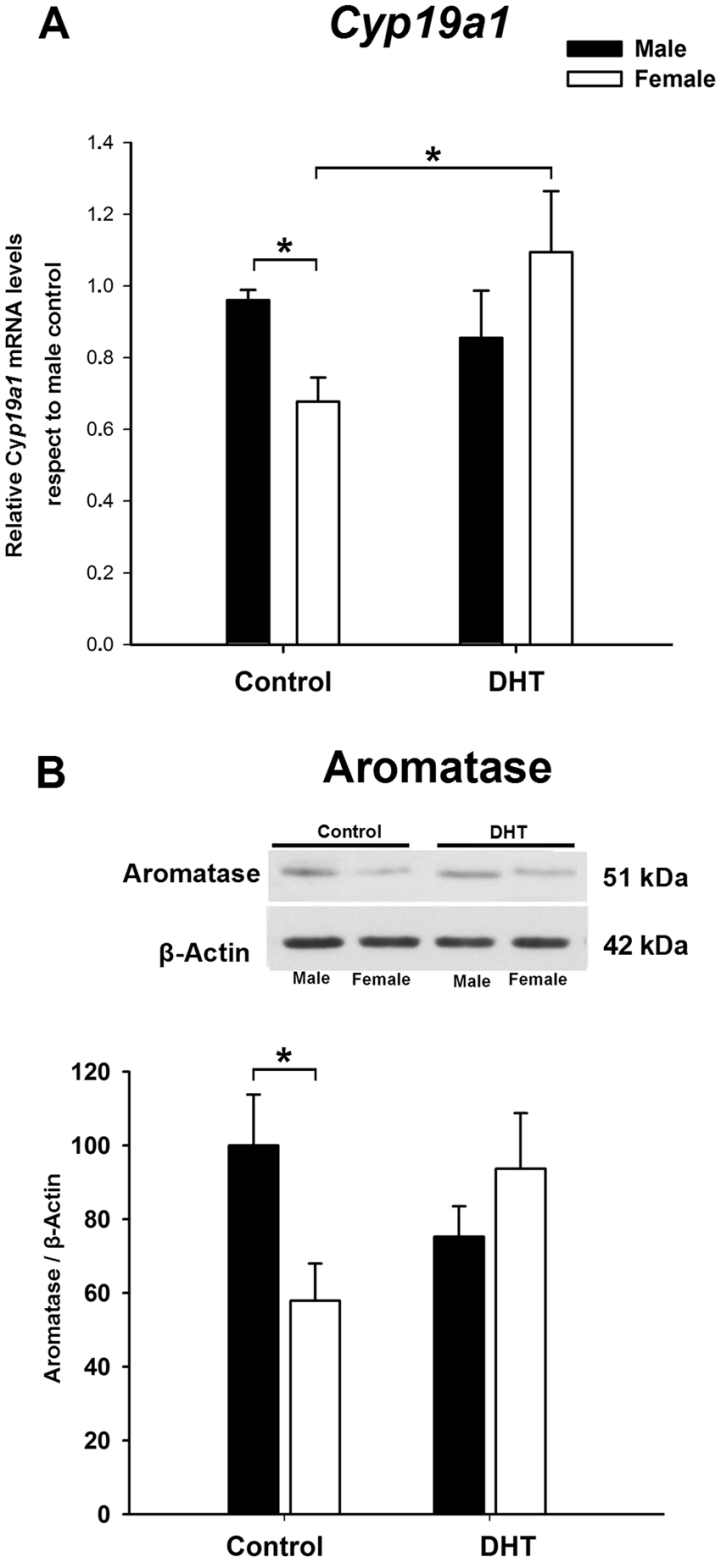
Effect of dihydrotestosterone (DHT) on aromatase mRNA (*Cyp19a1*, **A**) and protein (**B**) in male and female primary amygdala neuronal cultures from CD1 mouse embryos. DHT stimulation increased aromatase mRNA levels in females but not in male cultures. *p ≤ 0.05. Bars indicate mean + SEM. *n* = 4–6 independent cultures.

We first assessed the effect of DHT in male and female amygdala neuronal cultures. As expected, after DHT treatment *Cyp19a1* levels were increased in female cultures to male control levels (sex-by-treatment interaction: F_1,19_ = 6.56; p = 0.019; Fig. 4A). DHT did not increase *Cyp19a1* levels in male neurons (p = 0.55). Similar results were obtained for aromatase protein levels (Fig. 4B). Two-way ANOVA revealed an interaction of sex by treatment (F_1,21_ = 5.18; p = 0.033; Fig. 4B) with the final outcome of an abolition of sex differences between male control and DHT-treated female cultures (LSD test: p = 0.74) without affecting the aromatase levels in male cultures (p = 0.15).

**Figure 5:**
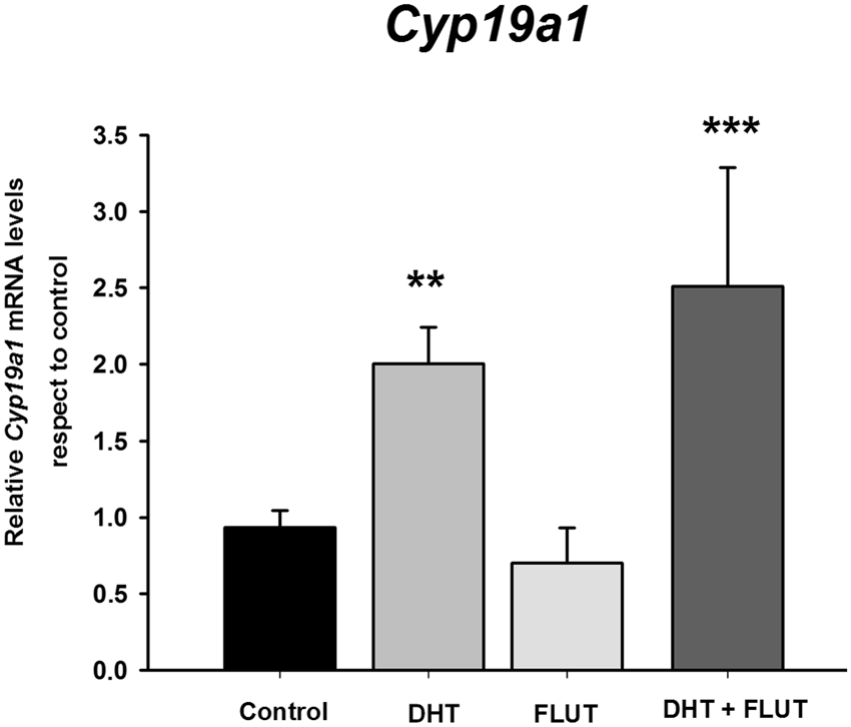
Effect of the selective anti-androgen flutamide on DHT-induced aromatase mRNA (*Cyp19a1*) expression in amygdala neuronal cultures from female CD1 mouse embryos. Flutamide did not prevent the effect of DHT on aromatase expression. **p < 0.01, ***p < 0.01. Bars indicate mean + SEM. n = 4–6 independent cultures.

To determine whether DHT regulates *Cyp19a1* through androgen receptors we assessed the effect of treatment with DHT in combination with the anti-androgen flutamide. We found a significant effect of treatment (ANOVA: F_3,19_ = 6.17; p = 0.004; Fig. 5), the androgen receptor antagonist flutamide did not block the effect of DHT on *Cyp19a1* levels (LSD test: p = 0.003). *Cyp19a1* levels in female cultures treated with DHT + flutamide remained high as in DHT alone (LSD test: p = 0.289).

**Figure 6:**
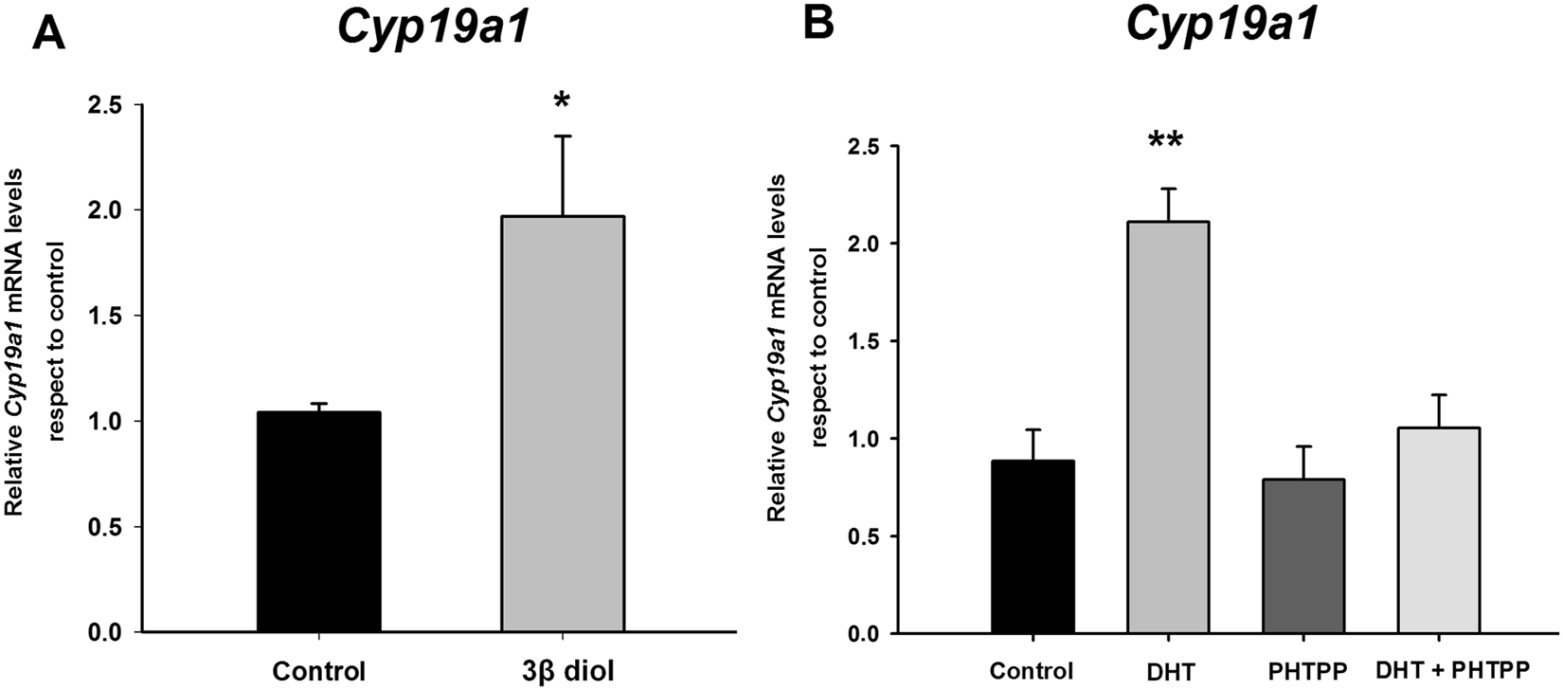
Effect of 5α-androstane-3β,17βdiol (3β-diol) (**A**) and the selective antagonist of ERβ PHTPP (**B**) on aromatase mRNA (*Cyp19a1*) in female amygdala neuronal cultures from CD1 mouse embryos. 3β-diol treatment increased *Cyp19a1*levels, while PHTPP blocked the effect of DHT on *Cyp19a1* levels. *p ≤ 0.05, **p < 0.01 vs. control. Bars indicate mean + SEM. *n* = 4–6 independent cultures.

DHT is a potent androgen involved in brain sexual masculinization not only through a direct activation of androgen receptors, but also indirectly via conversion to the ERβ agonist 3β-diol. Therefore, we tested the hypothesis that the regulation of aromatase by DHT is mediated by its metabolite 3β-diol. We first evaluated the direct effect of 3β-diol on aromatase expression in female amygdala cultures. Our results indicate that the ERβ agonist 3β-diol imitated the effect of DHT on aromatase expression, increasing *Cyp19a1* mRNA levels (Student’s t-test: p = 0.035; Fig. 6A). Furthermore, treatment of the cultures with the ERβ antagonist PHTPP completely blocked the effect of DHT on *Cyp19a1* expression (one-way ANOVA: F_3,14_ = 3.98; p = 0.03; LSD test: p = 0.045 for DHT vs. DHT + PHTPP; Fig. 6B). These findings suggest that DHT abolishes the sex difference in aromatase expression in amygdala neurons, increasing *Cyp19a1* mRNA levels in female neurons by a mechanism involving its metabolite 3β-diol and ERβ.

### Male neurons from amygdala expressed higher levels of ERβ mRNA than female neurons

**Figure 7:**
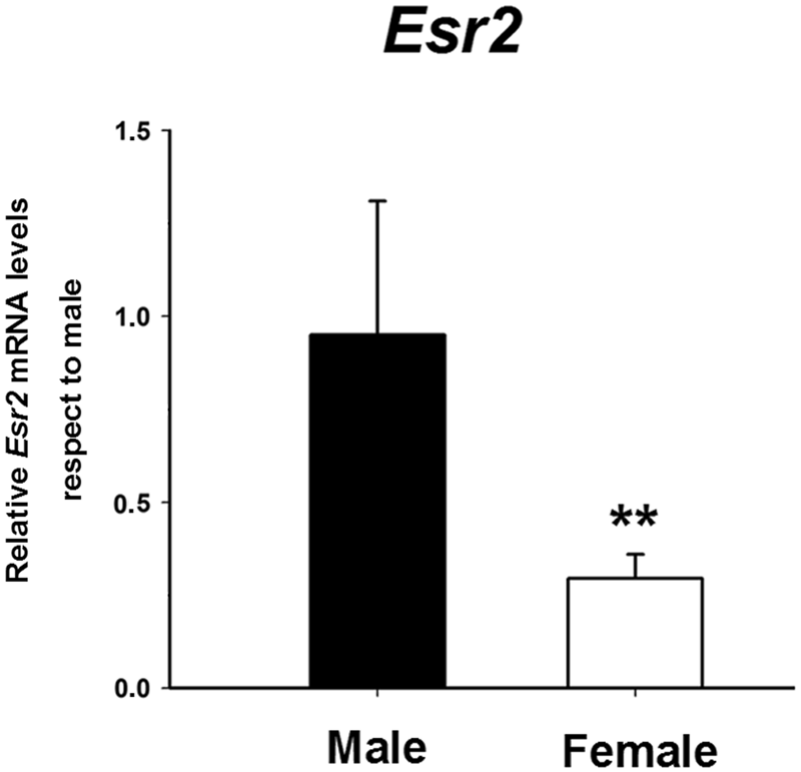
Expression of ERβ mRNA (*Ers2*) in male and female primary amygdala neuronal cultures from CD1 mouse embryos. Male cultures had higher expression of *Ers2* than females. **p ≤ 0.01. Bars indicate mean + SEM. *n* = 4–6 independent cultures.

In order to determine whether regulation of aromatase in amygdala neurons is secondary to a differential availability of ERβ between sexes we evaluated the mRNA expression of *Esr2* in male and female cultures. We found a clear sex difference with a significant lower expression of *Esr2* in female neurons (Student’s t-test: p = 0.01; Fig. 7).

### E2 and DHT increased ERβ expression in neurons from XX male and XX female embryos of the Four Core Genotype (FCG) mouse model

**Figure 8:**
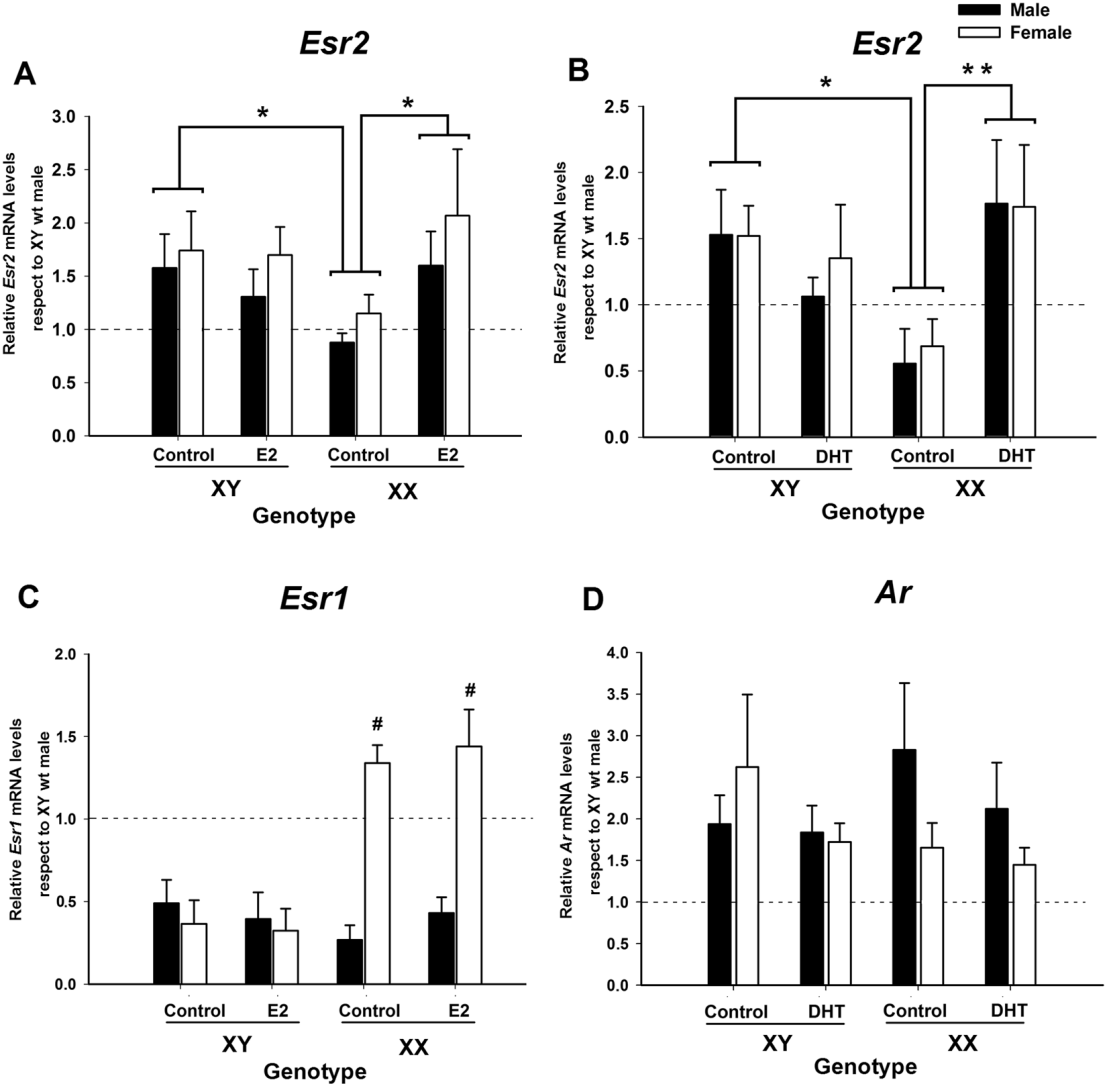
Effect of oestradiol (E2) on *Ers2* (**A**) and *Ers1* (**C**) levels or dihydrotestosterone (DHT) on *Ers2* (**B**) and *Ar* (**D**) levels in amygdala neuronal cultures of FCG mouse model. E2 and DHT treatments increased *Ers2* levels in XX cultures but not in XY cultures. Hormonal treatments did not affect *Esr1* and *Ar* among genotypes. *p < 0.05, **p < 0.01, ^#^p < 0.001 (LSD Fisher). Bars indicate mean + SEM. *n* = 4–6 independent cultures for each genotype and hormone treatment. Embryos were obtained from 5 pregnant mothers.

The results above demonstrate that E2 and DHT regulated aromatase expression only in female neurons through a mechanism involving ERβ activation. We therefore asked whether the sex chromosome complement that determines the capacity for aromatase regulation is associated to the regulation of ERβ in XX neurons. To deal with this question, ERβ expression was assessed in amygdala neuronal cultures from E15 embryos belonging to FCG mice. As shown in Fig. 8, E2 treatment of FCG neuronal cultures resulted in a significant increase in *Esr2* expression in neurons derived from either XX male or XX female embryos, but not in XY neurons of either gonadal sex. Three-way ANOVA in cultures treated with E2 revealed a genotype-by-hormone interaction (F_1,25_ = 4.53; p = 0.043; Fig. 8A). Treatment with E2 in XY neurons was not able to regulate *Esr2* levels (LSD test: p = 0.58) while in XX neurons *Esr2* expression was significantly increased (p = 0.027) with an overall effect of abolishment of genotype-induced sex differences between XX and XY neurons (p = 0.69). Interestingly, FCG neuronal cultures stimulated with DHT demonstrated a similar regulation for ERβ mRNA. DHT treatment resulted in a genotype-by-treatment interaction in the ANOVA (F_1,27_ = 7.24; p = 0.012; Fig. 8B). DHT stimulation resulted in a significant increment in *Esr2* levels in XX cultures (p = 0.01) but not in XY cultures (p = 0.38). Thus, both hormonal treatments counteracted the sex chromosome effect making XX cultures treated with E2 or DHT equivalent to XY control cultures.

We also assessed the influence of sex chromosome factors on the expression of ERα and AR in amygdala neuronal cultures. Three-way ANOVA showed a significant main effect of gonadal sex (Sry gene; F_1,31_ = 18.31; p = 0.0002), sex chromosome complement (F_1,31_ = 18.65; p = 0.0001), and interaction of both factors (F_1,31_ = 26.65; p = 0.00001) on *Esr1* expression. XX female cultures showed higher expression levels of *Esr1* and E2 stimulation did not significantly change *Esr1* mRNA (Fig. 8C). The analysis of *Ar* expression showed no effect of hormone treatment, gonadal status, or sex chromosome factors (Fig. 8D).

## Discussion

Previously we have demonstrated a sex difference in aromatase expression in the anterior amygdaloid area of the MF1 FCG mouse brain and culture^7^. In order to corroborate the existence of this difference in wild-type mice, we used CD1 mice since this strain is commonly used to prepare primary neuronal cultures. In agreement with previous data in MF1 FCG mice^7^, male neuronal cultures from CD1 embryos expressed higher levels of *Cyp19a1* mRNA. Furthermore, our present data also show similar sex differences in *Esr2* expression in cultures from MF1 and CD1 mice. Therefore, although the use in the present study of two different mice strains, MF1 and CD1, is not exempt of some limitations, our findings suggest that both strains have similar characteristics concerning aromatase and ERβ expression in the amygdala and that CD1 mice is a valid model to study the mechanism of hormonal regulation of aromatase *in vitro*.

We have found a novel mechanism by which sex chromosomes orchestrate sex differentiation in non-gonadal tissues. In concordance with our previous results we found that aromatase is differentially expressed and regulated according to the sex chromosome complement of mouse amygdala neurons. Furthermore, our present results demonstrate that XX amygdala neurons are sensitive to E2 or DHT, thereby increasing ERβ and aromatase levels. We found that in female amygdala neurons, E2, DHT and the ERβ agonist DPN increased aromatase expression. Flutamide was not able to prevent aromatase regulation by DHT, while the ERβ antagonist PHTPP blocked the effect of both E2 and DHT. In addition, 3β-diol, which has been reported to preferentially bind ERβ^27^, mimics effects of E2 and DHT on aromatase expression. Remarkably our present results, in concordance with previous findings^7^, demonstrate that this mechanism is only possible in neurons derived from XX embryos, independently of their gonadal status.

Multiple previous studies in rodents have investigated aromatase levels in the brain; however, few have taken into account the expression of aromatase prior to the organizational actions of gonadal hormones. Exceptions are works from Beyer^8, 9^ and Negri-Cesi^11^ which reported a higher activity and expression of aromatase in the hypothalamus in male brain at E16. In the present study, we found a sex difference in aromatase expression in amygdala neurons of E15 embryos. Male cultures showed higher aromatase mRNA and protein levels than females. To our knowledge, this is the first report of sex differences in aromatase expression in amygdala region as early as embryonic day E15 in mouse.

Regulation of brain aromatase by gonadal hormones in rodents has been demonstrated previously^18–22^. Studying aromatase mRNA expression in different brain regions of the adult male and female rat, Tabatadze *et al*.^28^ reported that the amygdala was the only brain region in which both sex and hormones affect aromatase expression (males > females). They showed that gonadal/hormonal upregulation of aromatase expression occurred in both sexes, whereas in our study only females respond to E2 or DHT with augmented levels of aromatase suggesting differential hormonal sensitivity of amygdala between prenatal period and adulthood. Although our findings show an all or none sex dimorphic response to E2 and DHT, it is important to mention that we have assessed only a single time point (2 h post-treatment) and a single dose of the hormones. Subtler differences in the sensitivity of male and female amygdala neurons to E2 and DHT may be revealed by assessing additional doses and time points.

There are several possible explanations for the different response of male and female neurons to exogenous hormones. One important consideration is that amygdala regions are rich in nuclear AR and ERs^25, 29^ that could be expressed differently in both sexes at E15–16. Previously we have shown a higher AR mRNA expression in the anterior amygdala of male mice, compared to females, at E16^7^. Remarkably, the expression of ERα mRNA in the same region was higher in female than in male mice^7^. In developing rats ERα mRNA levels are present in preoptic and amygdala regions since E18^30^. In addition, it was described that ERα and ERβ are expressed in posterodorsal part of the medial amygdaloid nucleus during early postnatal development^31^. In order to unravel the participation of classical and non-classical steroid receptor-dependent mechanisms we assessed several compounds that act as agonists or antagonists of AR and ERs. DPN, an ERβ agonist, imitated the effect of E2 on aromatase expression. Moreover, the effects of E2 were blocked by co-administration of the ERβ antagonist, PHTPP. By contrast, PPT and G1, selective agonists of ERα and GPER, respectively, were not able to imitate the effect of E2 on aromatase expression. Furthermore, the effect of DHT on aromatase was not antagonized by flutamide, a selective AR antagonist; but was completely blocked by PHTPP, the selective antagonist of nuclear ERβ. All these findings suggest that the hormones E2 and DHT are regulating aromatase expression by a classical nuclear ERβ mediated transcription.

Based on these results, we directly evaluated ERβ mRNA in our sexually segregated amygdala culture system. Surprisingly, we found a sex difference in ERβ mRNA expression in favour of males. This finding appears to be counterintuitive to results demonstrating that only female neurons were responsive to hormone treatments. One possible explanation for this apparent contradiction is that ERβ may be under different hormonal regulation in males and females. To address this issue we evaluated ERβ mRNA in amygdala cultures of FCG mouse model treated with E2 or DHT. Neurons with the XY sex chromosomes showed higher ERβ expression levels than neurons with XX sex chromosomes, irrespective of gonadal type. Hormonal treatments increased ERβ only in XX, resulting in the abolishment of sex differences in ERβ expression by a mechanism that also depends on genetic factors. A complementary and/or alternative explanation is that, in males, E2 may be degraded into catechol-oestrogens via hydroxylation by aromatase^32^ which would lead to a sexually differential regulation of the enzyme itself. Although it was not possible to assess ERβ protein levels, due to the lack of adequate antibodies^33^, our study strongly supports that ERβ participates in the regulation of aromatase in amygdala at E15.

DHT, a 5α-reduced form of testosterone, has been classically considered a prototypical AR agonist with no ability to be aromatized to oestrogen-like metabolites. However, several reports reviewed by Handa *et al*.^34, 35^ indicate that DHT, in a fashion similar to T, may be converted to products with oestrogen-like activity by other enzymes than aromatase. One of these products is 3β-diol which preferentially binds to ERβ, and not to AR, often through a canonical ERE in the promoter of a given target gene^36^. Here we have found that the increase in aromatase expression by DHT could be blocked by co-administration of the ERβ antagonist, PHTPP, but not by the AR antagonist, flutamide. Furthermore, 3β-diol treatment to amygdala female cultures increased aromatase levels. Together, these results provide correlative evidence that 3β-diol mediates the effects of DHT on aromatase expression by binding ERβ. These findings may also explain the contradictory results found by other groups in amygdala region in which DHT seems to be more effective than T in the induction of aromatase^21^. Harada *et al*.^23^ have demonstrated that androgens and oestrogens synergize to induce aromatase mRNA in the quail brain. Also in the preoptic area and hypothalamus of mouse brain T and DHT induce brain aromatase expression^24^. This endocrine pathway has been shown to exist in numerous species and tissues^37^, but its functional significance was first suggested for prostate^38^, where it has been proposed that 3β-diol is the predominant endogenous oestrogen. In teleost fish, the regulation of the cyp19a1b gene by DHT also involves its conversion into 3β-diol^39^. The fact that gonadal/hormonal up regulation of aromatase depends on ERβ parsimoniously explains why E2 and DHT produce the same effect on aromatase expression.

The aromatase gene contains androgen and oestrogen response elements^23, 40, 41^ suggesting that its regulation is through classical steroid receptor-dependent mechanisms. Consistent with this, results from Pak *et al*.^36^ show that 3β-diol significantly increased ERE-mediated promoter activity to levels greater than that of E2, whereas it had no effect at AP-1 site. Yilmaz *et al*.^42^ using a hypothalamic neuronal cell line demonstrated that E2 positively regulates the brain-specific promoter of aromatase gene via a mechanism involving ERα. Our present results in amygdala neurons of developing mouse brain indicate that androgens and oestrogens increase aromatase expression through a common mechanism that involves the action of 3β-diol and the activation of ERβ, highlighting the complex region-specific mechanism that regulates aromatase expression in the brain.

**Figure 9:**
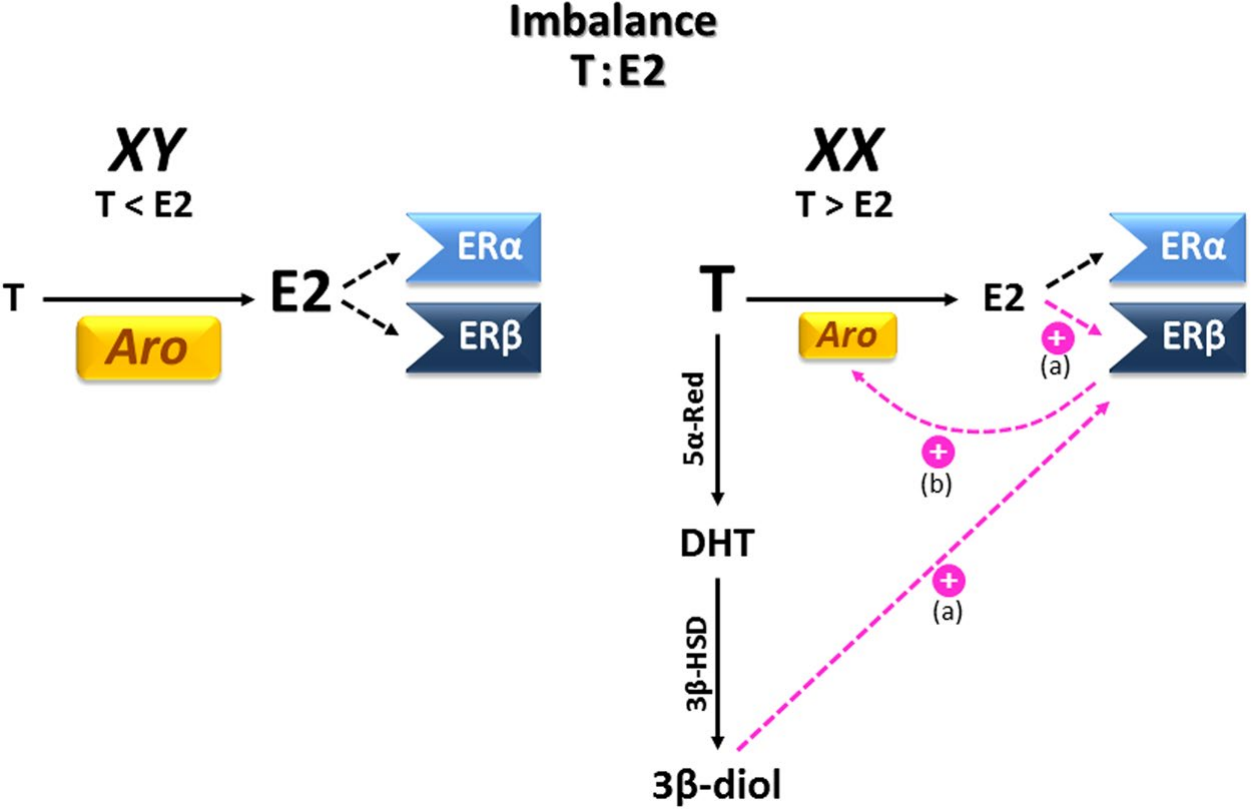
Summary of the effects of sex steroids in aromatase and ERβ expression in XX and XY amygdala neurons. Differential expression of aromatase produces an imbalance in the E2:T ratio favouring E2 action in XY and T action in XX. In XY amygdala high levels of aromatase may favour E2 action (E2 > T) through ERα/β. On the other hand, in XX amygdala low levels of aromatase may promote T signalling (T > E2) which is then converted by 5α-reductase to DHT. DHT is further metabolized to 3β-diol that preferentially binds more to ERβ than to ERα. E2 or DHT exposure increased ERβ expression only in XX amygdala cultures (**a**). Thus, the predominance of ERβ activation in XX, over ERα in XY, increases aromatase expression only in XX (**b**). The final result is the cancellation of sex differences in aromatase expression ensuring equivalent levels of neurosteroidogenesis in male and female amygdala before the critical period of brain development.

While a majority of studies in the brain have focused on sexual dimorphic expression and hormonal modulation of aromatase, other factors controlling aromatase expression during early development have been less studied. We have previously shown that sex chromosome complement induces sex differences in aromatase expression and regulation in limbic regions of the embryonic mouse brain^7^. In this study, we have found that hormonal regulation of aromatase in amygdala neurons depends on ERβ signalling. It could be hypothesized that the differential aromatase expression produces an imbalance in E2:T ratio between XY/XX brain amygdala during development (Fig. 9). In XY amygdala high levels of aromatase may favour E2 action (E2 > T) through ERα/β. On the other hand, in XX amygdala low levels of aromatase may promote T signalling (T > E2) which is then converted by 5α-reductase to DHT. DHT is further metabolized to 3β-diol that preferentially binds more to ERβ than to ERα. By means of the FCG model, we have described a differential expression of ERβ mRNA between XX and XY amygdala cultures. Furthermore, our results showed E2 or DHT exposure increased ERβ only in XX amygdala cultures. Thus, the predominance of ERβ activation in XX, over ERα in XY, increases aromatase expression only in XX. The final result is the compensation of the sex differences in aromatase expression ensuring equivalent levels of neurosteroidogenesis in male and female amygdala before the sensitive period of brain development.

The sex chromosome-induced sex differences found for ERβ expression in amygdala neurons could be interpreted as an additional mechanism by which sex chromosomes regulate aromatase expression ensuring adequate levels of ERβ in XX neurons for aromatase regulation during development. The fact that ERα and AR expressions were not subjected to hormonal regulation supports this interpretation. The regulation of both, *Cyp19a1* and *Esr2*, autosomal genes could be the consequence of the differential expression of X/Y-linked genes produced by the imbalance (in number or type) of sex chromosomes in males and females. The FCG mouse model allowed us to preclude the effect of Sry gene on the expression of ERβ since we found a significant difference between XX male (Sry+) neurons compared with XY male (Sry+) neurons. However, other Y genes have an inherently male function because they act on germ cells in a cell-autonomous fashion and are required for spermatogenesis; a male-specific function^43^. The latter could be interpreted as a possible explanation in the significant differences found between XY female and XX female neurons. It could also be possible that differences in the number of X chromosomes cause the sex differences in aromatase and ERβ expression. As it was recently reviewed by Arnold^3^, sex differences in gene expression also arise due to the female specific expression of Xist, a higher expression of X escapee genes in XX than in XY cells, the sexual inequality of parental imprints on X chromosome genes or due to female-specific effects of the inactive X chromosome. Some genes present in X or Y chromosomes encode for transcriptional regulatory proteins which are able to produce changes in chromatin remodelling and regulate autosomal gene expression^44^. In line with this, Nugent *et al*.^45^, have reported that aromatase gene *Cyp19a1* is subjected to a sex-related differential DNA methylation in rat preoptic area during the critical period of sexual differentiation. Also the DNA methylation in CpG islands present in the promoter region of ERβ rat gene was reported to be differentially regulated across the life span in a manner specific to the brain region, age, sex, and neonatal hormone exposure of the animal^46^.

Sex specific regulation of aromatase expression reported here and in our previous study^7^ could be related to a neuroprotective mechanism evolved to ensure adequate levels of aromatase in XY brain during development. As it was previously stated, the autism spectrum disorder, a sex-biased neurodevelopmental disorder which occurs two to four times more often in boys and men than in females (reviewed in Romano *et al*.^47^) has been related to environmental factors, especially the intrauterine developmental environment. Results from Xu *et al*.^48^ indicate that the prenatal hyperandrogenic environment produced by letrozol treatment induces autistic-like behaviour in rat offspring. Future studies could explore the upstream pathways involved in this mechanism.

In summary, we have demonstrated that hormonal and genetic factors contribute to regulate aromatase expression in amygdala neurons before the critical period of brain masculinization. Our findings propose a novel mechanism orchestrated by sex chromosomes in which ERβ (but not ERα or AR) acts as the final effector in the action of E2 and DHT on aromatase expression in developing amygdala neurons.

## Methods

### Animals

The embryos were obtained from CD1 mice raised in the Cajal Institute (Madrid, Spain) (Figs 1–4 and 7) and in the Ferreyra Institute (Córdoba, Argentina) (Figs 5 and 6). Furthermore, some experiments were performed with MF1 “Four Core Genotypes” (FCG) mice born and reared in the Ferreyra Institute (Córdoba, Argentina) (Fig. 8). The embryonic day 1 (E1) was defined as the day of vaginal plug. All procedures for handling and sacrificing the animals used in this study were approved by the animal care and use committees at our institutions and by the Consejeria del Medio Ambiente y Territorio (Comunidad de Madrid, Ref. PROEX 200/14) and followed the NIH guidelines for the care and use of laboratory animals. Genotyping was performed as previously described^7^.

### Primary amygdala neuronal cultures and treatments

E15 mouse embryos were classified according to the sex or/and genotype. Cultured amygdala neurons were prepared and maintained as described^7^. The medium was phenol red-free Neurobasal supplemented with B-27, N-2 and GlutaMAX I (Invitrogen). After 3 DIV the culture medium was replaced for 2 h by fresh medium devoid of supplements for Western blotting and quantitative real-time polymerase chain reaction analysis. Neuronal cultures were treated for 2 h with the following test compounds alone or in combination: E2 (10^−10^ M; Sigma-Aldrich), DHT (10^−10^ M; Steraloids), 3β-diol (10^−10^ M; Sigma-Aldrich), the selective ERα agonist propylpyrazoletriol (PPT, 10^−7^ M; Tocris Bioscience), the selective ERβ agonist dyarilpropionitrile (DPN, 10^−9^ M; Tocris), the GPER agonist G1 (10^−7^ M, Calbiochem), the selective ERβ antagonist 4-[2-phenyl-5,7-bis(trifluoromethyl)pyrazolo[1,5-a]pyrimidin-3-yl]phenol (PHTPP, 10^−9^ M; Tocris) and the selective AR antagonist flutamide (10^−7^ M; Tocris).

### RNA purification and qRT-PCR

Total RNA was isolated from neuronal cultures with TRIZOL reagent (Invitrogen) as directed by the manufacturer and quantified using NanoDrop spectrometer (Thermo Scientific). The RNA was reverse-transcribed into cDNA using random primers (Biodynamics) and SuperScript II reverse transcriptase (Invitrogen). *Cyp19a1*, *Ar*, *Esr1* and *Esr2* gene expression were determined using SYBR Green Real-Time PCR in the Step One^TM^ Real Time PCR System (Applied Biosystems). The efficiency of the amplification was established using a calibration curve (5-point 1:3 dilutions) and the dilution used for the samples was 1:3 from the initial concentration. All primer-pairs showed a single product in the melting curve analysis and efficiencies [E = 2 ± 0.1 (E = 10^[−1/slope]^)] between 90 to 110% (Table 1). For each sample, the Ct was determined and normalized to the average of the housekeeping gene 18 s rRNA. All samples were run in duplicate with the average Ct used for each sample. Relative quantifications of mRNA levels were determined by the ΔΔCt method except for *Cyp19a1* (E = 2.15) that was analysed according to Pfaffl^49^ [ratio = (E^target^)^∆Ct(target)^/(E^18S^)^∆Ct(18S)^, where E is the efficiency of primers and ∆Ct = Ct_(control)_ −Ct_(experimental)_].

**Table 1:** Primer sequences and conditions of qPCR assay.

Gene	GenBank	Primer Sequence (forward and reverse)	Primer Concentration (nM)	Product Size (bp)	Location relative to GenBank	Efficiency (%)	No. of Cycles (std.curve)
Cyp19a1	NM_007810.4	F:CGGGCTACGTGGATGTGTT	800	135	586F	115	26–31.5
		R:GAGCTTGCCAGGCGTTAAAG			720R		
Esr1	NM_007956.4	F:ATGAAAGGCGGCATACGGAAAG	200	94	944F	97	30–34
		R:CACCCATTTCATTTCGGCCTTC			1037R		
Esr2	NM_010157.3	F:CCTGGTCTGGGTGATTTCGA	800	100	1712F	90	29–35
		R:ACTGATGTGCCTGACATGAGAAAG			1811R		
Ar	NM_013476.3	F:GGCGGTCCTTCACTAATGTCAACT	800	84	2224F	102	29–33
		R:GAGACTTGTGCATGCGGTACTCAT			2307R		
18SrRNA	NR_003278.3	F:CGCCGCTAGAGGTGAAATTCT	500	67	949F	94	10–13
		R:CATTCTTGGCAAATGCTTTCG			1015R		

### Western blot

Western blotting was performed on homogenates of amygdala neurons raised in culture medium immediately after cell treatment. Cultures were washed twice with PBS at 37 °C and scraped with a rubber policeman into Ripa’s buffer (150 mMNaCl, 0.1% NP40, 0.5% sodium deoxycholate, 0.1% sodium dodecyl sulfate (SDS), 50 mMTris, pH 7.5) with protease inhibitors (1 μg/ml aprotinin, 1 μg/ml leupeptin, 1 μg/ml pepstatin A, and 100 μg/ml PMSF, 1 mM NaVO_4_). Aliquots of each sample were used for total protein quantification according to Lowry *et al*.^50^. The samples were homogenized, combined with an equal volume of Laemmli’s buffer (4% SDS, 20% glycerol, 10% β-mercaptoethanol, 125 mMTris, pH 6.8), boiled at 100 °C for 5 min, and centrifuged at 10,000 g for 15 min. Protein samples (20 μg/lane) were separated by 10% SDS-PAGE and transferred onto polyvinylidene fluoride membranes (Bio-Rad, Hercules, CA). Rabbit anti-aromatase antibody (diluted 1:1000) is directed to amino acids 488–502 of mouse aromatase^51^. The aromatase antibody resulted in the staining of two bands, including a band at 51 KDa corresponding to the molecular weight of aromatase^52^. Secondary antibody conjugated to horseradish peroxidase (Jackson, West Grove, PA) was used for the detection by enhanced chemiluminescence on X-ray film. After aromatase antibody blots were stripped by washing 10 times for 5 min each with Tris-buffered saline (TBS)-Tween (0.1%) at room temperature, 30 min at 55 °C with stripping buffer (62.5 mMTris-HCl, pH 6.8, 2% SDS, 100 mMβ-mercaptoethanol), and finally 10 times for 5 min each with TBS-Tween at room temperature. The stripped blots were then reproved with a monoclonal antibody against β-actin (Sigma A5316, diluted 1:2500) as loading control. The resulting film samples were scanned and analysed with the ImageJ program (freely available at imagej.nih.gov).

### Statistical analysis

Relative gene or protein expression was assessed by two-way ANOVA (gonadal sex × hormonal treatment) or three-way ANOVA (sex chromosome complement × gonadal sex × hormonal treatment). The loci of significant interactions were further analysed by Fisher’s least significance difference (LSD) post-hoc. When there were only two experimental groups, data was analysed using the Student’s t-test. A level of p < 0.05 was considered statistically significant. The n for statistical analysis was the number of independent neuronal cultures and corresponds to the number of pregnant mothers in CD1 cultures. For cell cultures prepared from FCG mice the number of independent neuronal cultures corresponds to the number of embryos of each genotype and treatment. The FCG embryos were obtained from 5 pregnant mothers.
